# Self-assembling nanoparticles encapsulating zoledronic acid revert multidrug resistance in cancer cells

**DOI:** 10.18632/oncotarget.5058

**Published:** 2015-09-09

**Authors:** Joanna Kopecka, Stefania Porto, Sara Lusa, Elena Gazzano, Giuseppina Salzano, Antonio Giordano, Vincenzo Desiderio, Dario Ghigo, Michele Caraglia, Giuseppe De Rosa, Chiara Riganti

**Affiliations:** ^1^ Department of Oncology, University of Torino, Torino, Italy; ^2^ Department of Biochemistry, Biophysics and General Pathology, Second University of Naples, Naples, Italy; ^3^ Department of Pharmacy, Federico II University of Naples, Naples, Italy; ^4^ Center for Pharmaceutical Biotechnology and Nanomedicine, Northeastern University, Boston, MA, USA; ^5^ Sbarro Institute for Cancer Research and Molecular Medicine, Center for Biotechnology, College of Science and Technology, Temple University, Philadelphia, PA, USA; ^6^ Department of Medicine, Surgery and Neuroscience University of Siena, Italy; ^7^ Department of Experimental Medicine, Second University of Naples, Naples, Italy

**Keywords:** self-assembling nanoparticles, zoledronic acid, ATP binding cassette transporters, multidrug resistance, hypoxia inducible factor-1α

## Abstract

The overexpression of ATP binding cassette (ABC) transporters makes tumor cells simultaneously resistant to several cytotoxic drugs. Impairing the energy metabolism of multidrug resistant (MDR) cells is a promising chemosensitizing strategy, but many metabolic modifiers are too toxic *in vivo*. We previously observed that the aminobisphosphonate zoledronic acid inhibits the activity of hypoxia inducible factor-1α (HIF-1α), a master regulator of cancer cell metabolism. Free zoledronic acid, however, reaches low intratumor concentration. We synthesized nanoparticle formulations of the aminobisphosphonate that allow a higher intratumor delivery of the drug. We investigated whether they are effective metabolic modifiers and chemosensitizing agents against human MDR cancer cells *in vitro* and *in vivo*.

At not toxic dosage, nanoparticles carrying zoledronic acid chemosensitized MDR cells to a broad spectrum of cytotoxic drugs, independently of the type of ABC transporters expressed. The nanoparticles inhibited the isoprenoid synthesis and the Ras/ERK1/2-driven activation of HIF-1α, decreased the transcription and activity of glycolytic enzymes, the glucose flux through the glycolysis and tricarboxylic acid cycle, the electron flux through the mitochondrial respiratory chain, the synthesis of ATP. So doing, they lowered the ATP-dependent activity of ABC transporters, increasing the chemotherapy efficacy *in vitro* and *in vivo*. These effects were more pronounced in MDR cells than in chemosensitive ones and were due to the inhibition of farnesyl pyrophosphate synthase (FPPS), as demonstrated in *FPPS*-silenced tumors.

Our work proposes nanoparticle formulations of zoledronic acid as the first not toxic metabolic modifiers, effective against MDR tumors.

## INTRODUCTION

The mevalonate pathway produces cholesterol and isoprenoids - such as farnesyl pyrophosphate (FPP) and geranylgeranyl pyrophosphate - which activate small G-proteins like Ras and Rho. The high activity of the mevalonate pathway induces tumor proliferation, invasion and aggressiveness, and is correlated with poor clinical outcome of oncological patients [1, 2]. Hence, inhibitors of the pathway are attractive adjuvant anti-tumor drugs [3–5].

The overexpression of ATP binding cassette (ABC) transporters - such as P-glycoprotein (Pgp/ABCB1), multidrug resistance related proteins (MRPs/ABCCs) and breast cancer resistance protein (BCRP/ABCG2) - limits the intracellular retention and activity of several cytotoxic drugs, producing a multidrug resistant (MDR) phenotype in tumor cells [6]. MDR cells have a higher mevalonate pathway than chemosensitive ones [7, 8]. Since the activity of ABC transporters is increased by the high content of cholesterol in the plasma membrane [7–9], the inhibition of mevalonate pathway has efficiently overcome the MDR phenotype *in vitro* [7–13].

To achieve the maximal efficacy, ABC transporters have also a huge need of ATP [6]. ATP depleting agents exert higher cytotoxicity against MDR cells than against chemosensitive ones, inducing a phenomenon known as “collateral sensitivity” [14, 15]. Although ATP depleting agents are very effective *in vitro*, they are too toxic *in vivo* [16].

Zoledronic acid (ZA), a clinically used aminobisphosphonate that inhibits the FPP synthase (FPPS) step in the mevalonate pathway [17], reduces the activity and expression of Pgp in MDR cells by decreasing the amount of cholesterol in plasma membrane and inhibiting the *Pgp* transcription mediated by the hypoxia inducible factor-1α (HIF-1α) [8]. Of note, HIF-1α also increases the energy metabolism and ATP synthesis in cancer cells [18].

The major drawback of using ZA at clinically achievable concentrations is its fast uptake by bone tissue that limits the amount of the drug reaching the tumor [19]. In previous studies we demonstrated that ZA has a negligible effect on different tumors *in vivo*, in consequence of its low intratumor accumulation. The use of nanocarriers such as nanoparticles (NPs) or liposomes made ZA a powerful anticancer agent by improving its intratumor delivery [20–24]. The use of nanovectors have enhanced the anti-proliferative activity of ZA in a wide spectrum of chemosensitive tumors [20–23], but it is not known whether NPs carrying ZA (here termed NZ) are also effective against MDR tumors.

In this work we demonstrated that NZ is a strong chemosensitizing agent, owing to its peculiar effects on the energy metabolism of MDR tumors.

## RESULTS

### NZ inhibits the mevalonate pathway/Ras/ERK1/2/HIF-1α/Pgp axis and sensitizes MDR cells to a broad spectrum of chemotherapeutic agents

We investigated the effects of ZA and NZ in non-small cell lung cancer A549 cells and in the chemoresistant counterpart A549/MDR cells, which had higher IC_50_ values towards different cytotoxic drugs (Table 1) and higher expression of different ABC transporters (Supplementary Figure 1). The NZ particles used in this study had a mean diameter of about 150 nm with a narrow size distribution (polydispersity index – P.I. – lower than 0.2). A deep characterization of these particles have been described in [20]. The IC_50_ of ZA, NZ and self-assembling nanoparticles without ZA (blank NPs) in A549 and A549/MDR cells are provided in the Table 2: on the basis of these values, in all the experiments we used ZA, NZ and blank NPs at the not toxic concentration of 1 μM.

**Table 1:** IC_50_ (μM) of different chemotherapeutic drugs in A549 and A549/MDR cells

		A549	A549	A549	A549/MDR	A549/MDR	A549/MDR
Drug	Transporter	CTRL	ZA	NZ	CTRL	ZA	NZ
**doxorubicin**	Pgp, MRP1, MRP2, MRP3, BCRP	0.53 ± 0.06	0.41 ± 0.07	0.36 ± 0.07 *	1.83 ± 0.14°	1.53 ± 0.12	1.01 ± 0.18 *
**vinblastine**	Pgp, MRP1, MRP2	2.34 ± 0.25	1.24 ± 0.12 *	0.78 ± 0.13 *	12.37 ± 0.21°	5.41 ± 0.64 *	1.86 ± 0.44 *
**etoposide**	Pgp, MRP1, MRP2, MRP3	3.25 ± 0.12	0.87 ± 0.14 *	0.51 ± 0.12 *	8.33 ± 0.44°	5.43 ± 0.27 *	0.91 ± 0.12 *
**irinotecan**	Pgp, MRP1, MRP2	4.21 ± 0.15	3.37 ± 0.12	2.11 ± 0.12 *	8.11 ± 0.42°	6.77 ± 0.43	3.33 ± 0.41 *
**cisplatin**	MRP1, MRP2, MRP4	8.11 ± 0.44	1.37 ± 0.09 *	0.81 ± 0.11 *	12.88 ± 0.21°	1.97 ± 0.27 *	0.65 ± 0.12 *
**oxaliplatin**	MRP1, MRP4	2.87 ± 0.14	1.34 ± 0.17 *	1.03 ± 0.12 *	5.20 ± 0.32°	3.21 ± 0.17 *	1.86 ± 0.31 *
**5-fluorouracile**	MRP1, MRP3, MRP4, MRP5	4.42 ± 0.18	4.11 ± 0.17	4.26 ± 0.09	7.01 ± 0.45°	5.88 ± 0.36	5.15 ± 0.12
**methotrexate**	MRP4, Pgp, MRP1, MRP2, MRP3, BCRP	3.27 ± 0.11	1.88 ± 0.28 *	0.61 ± 0.14 *	9.37 ± 0.63°	8.67 ± 1.09	4.32 ± 0.67 *
**pemetrexed**	MRP5	0.51 ± 0.11	0.25 ± 0.08 *	0.07 ± 0.02 *	11.21 ± 0.87°	8.76 ± 0.77	2.51 ± 0.47 *
**gemcitabine**	MRP5	0.07 ± 0.02	0.04 ± 0.01 *	0.03 ± 0.01 *	0.88 ± 0.15°	0.81 ± 0.13	0.12 ± 0.04 *
**mitoxantrone**	BCRP, Pgp, MRP1	10.22 ± 0.88	5.24 ± 0.67 *	1.17 ± 0.18 *	17.88 ± 0.67°	12.33 ± 0.73	2.67 ± 0.25 *

Untreated (CTRL) A549 and A549/MDR cells, cells treated with 1 μM ZA or NZ, were incubated for 72 h with increasing concentrations of chemotherapeutic drugs, then stained in quadruplicate with neutral red (*n* = 3). Versus respective CTRL: *p < 0.02; A549/MDR versus A549 cells: °*p* < 0.005.

**Table 2:** IC_50_ (μM) of ZA, NZ and blank NPs in A549 and A549/MDR cells

Sample	A549	A549/MDR
**ZA**	>120	89.62 ± 7.81
**NZ**	20.22 ± 1.23 *,°	20.78 ± 1.82 *,°
**NB**	>120	>120

A549 and A549/MDR cells were incubated for 72 h with increasing concentrations of ZA, NZ or blank NPs (NB), then stained in quadruplicate with neutral red (*n* = 3). Versus NB, in each cell line: *p < 0.001; versus ZA, in each cell line: °*p* < 0.001.

In A549/MDR cells NZ lowered the IC_50_ of different cytotoxic drugs, unrelated for structure, mechanism of action and efflux through specific ABC transporters, more than ZA (Table 1). Similar results were obtained in chemosensitive HT29 cells and in their resistant counterpart HT29/MDR cells (Supplementary Table 1). NZ and – at a lesser extent ZA – reduced the expression of Pgp, but did not change the levels of the other ABC transporters (Supplementary Figure 2).

**Figure 1:**
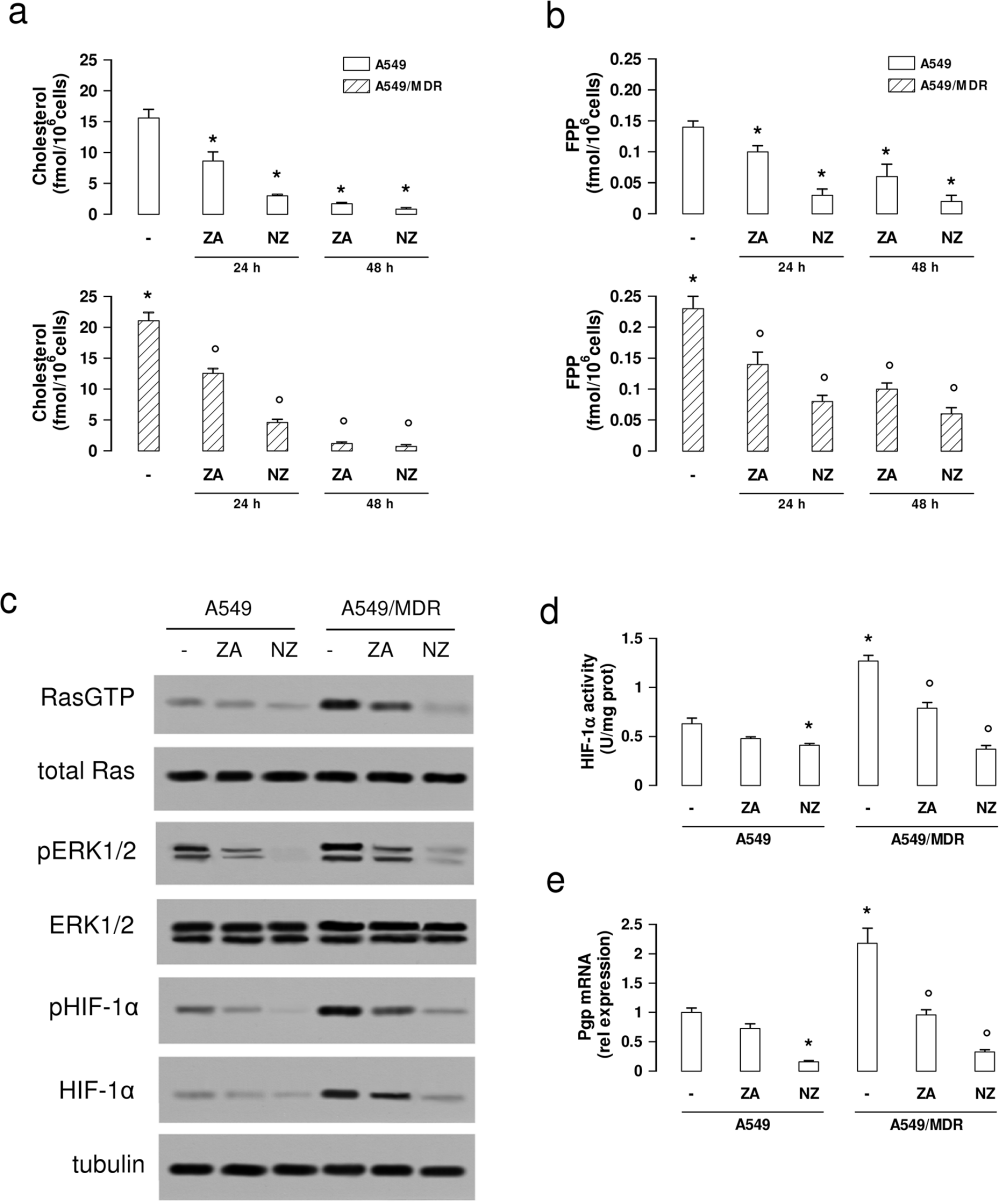
NZ lowers the mevalonate pathway/Ras/ERK1/2/HIF1α axis and Pgp expression in MDR cancer cells Chemosensitive human lung cancer A549 cells and their resistant counterpart A549/MDR cells were grown for 24 (panel a-b) or 48 h (panel a–e) in fresh medium (−), in medium containing 1 μM zoledronic acid (ZA) or 1 μM self-assembling ZA formulation (NZ). **a–b.** Cells were radiolabelled during the last 24 h with [^3^H]-acetate, then the *de novo* synthesis of cholesterol (panel a) or FPP (panel b) was measured. Data are presented as means ± SD (*n* = 3). For both panels, versus untreated A549 cells: *p < 0.05; versus untreated A549/MDR cells: °*p* < 0.005. **c.** Cells were lysed and subjected to the Western blot analysis for Ras-GTP, Ras, phospho(Thr202/Tyr204, Thr185/Tyr187)-ERK1/2, ERK1/2, phospho(Ser)-HIF-1α, HIF-1α. The β-tubulin expression was used as control of equal protein loading. The figure is representative of 3 experiments. **d.** HIF-1α activity was measured in nuclear extracts by ELISA. Data are presented as means ± SD (*n* = 4). Versus untreated A549 cells: *p < 0.05; versus untreated A549/MDR cells: ° *p* < 0.001. **e.** *Pgp* mRNA levels were detected in triplicate by qRT-PCR. Data are presented as means ± SD (*n* = 4). Versus untreated A549 cells: *p < 0.001; versus untreated A549/MDR cells: °*p* < 0.001.

We next analyzed if NZ reduced the mevalonate pathway activity, which favors the MDR phenotype and is inhibited by ZA [8]. NZ decreased the synthesis of cholesterol and FPP more than ZA, after 24 and 48 h; its effect was stronger in A549/MDR cells, which had a basally higher activity than A549 cells (Figure 1a–1b). In parallel, NZ lowered the activity of Ras and Ras-downstream effectors ERK1/2 (Figure 1c). HIF-1α, which was constitutively phosphorylated (Figure 1c) and bound to its DNA target sequence (Figure 1d) in A549/MDR cells, is a substrate of ERK [25]. NZ reduced the HIF-1α amount, phosphorylation and DNA binding (Figure 1c–1d), and lowered the transcription of the HIF-1α-target gene *Pgp* (Figure 1e) in MDR cells.

We next looked for potential mechanisms explaining the chemosensitizing effects of NZ on drugs that are not substrates of Pgp.

### By reducing HIF-1α activity, NZ decreases the glycolytic flux and the ATP levels in MDR cells

**Figure 2:**
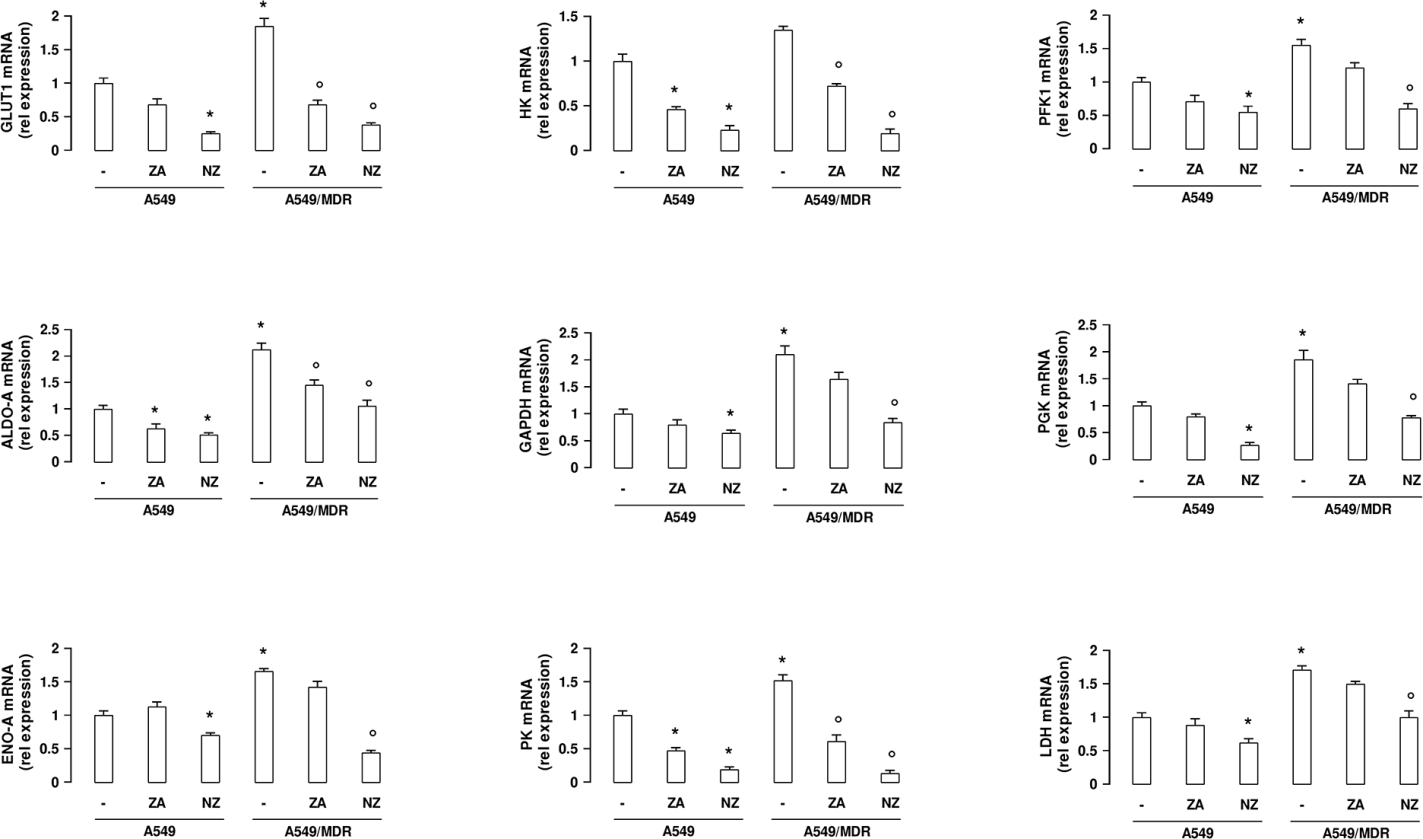
NZ reduces the expression of glycolytic genes in MDR cancer cells Chemosensitive human lung cancer A549 cells and their resistant counterpart A549/MDR cells were grown for 48 h in fresh medium (−), in medium containing 1 μM zoledronic acid (ZA) or 1 μM self-assembling ZA formulation (NZ). *GLUT1, HK, PFK1, ALDO-A, GAPDH, PGK, ENO-A, PK, LDH* mRNA levels were detected in triplicate by qRT-PCR. Data are presented as means ± SD (*n* = 4). For all panels, versus untreated A549 cells: *p < 0.05; versus untreated A549/MDR cells: ° *p* < 0.01.

Compared to A549 cells, A549/MDR cells had higher expression of the HIF-1α-target genes glucose transporter 1 (*GLUT1*), hexokinase (*HK*), phosphofructokinase-1 (*PFK1*), aldolase-A (*ALDO-A*), glyceraldehyde 3-phosphate dehydrogenase (*GAPDH*), phosphoglycerate kinase (*PGK*), enolase-A (*ENO-A*), pyruvate kinase (*PK*), lactate dehydrogenase (*LDH*; Figure 2), which are involved in glucose uptake and metabolism. NZ down-regulated all these genes (Figure 2), as well as other canonical HIF-1α-target genes, such as vascular endothelial growth factor, erythropoietin, carbonic anhydrase IX and XII (Supplementary Figure 3), in MDR cells.

**Figure 3:**
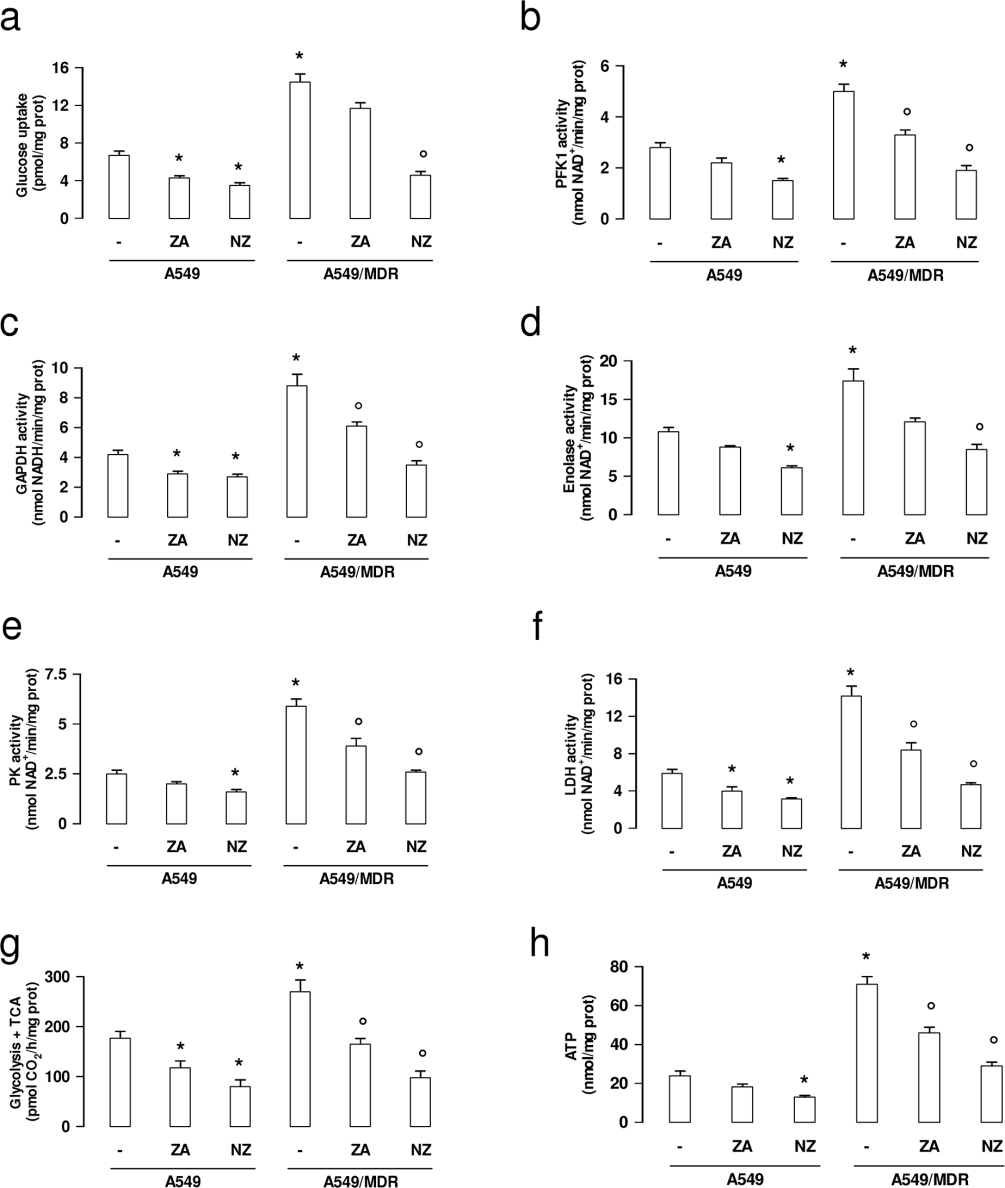
NZ decreases glucose uptake, glycolysis, tricarboxylic acid cycle, ATP levels in MDR cancer cells Chemosensitive human lung cancer A549 cells and their resistant counterpart A549/MDR cells were cultured for 48 h in fresh medium (−), in medium containing 1 μM zoledronic acid (ZA) or 1 μM self-assembling ZA formulation (NZ). **a.** The uptake of 2-deoxy-D-[^3^H]-glucose within living cells was measured and quantified by liquid scintillation. Data are presented as means ± SD (*n* = 3). Versus untreated A549 cells: *p < 0.002; versus untreated A549/MDR cells: °*p* < 0.001. **b–e.** The enzymatic activity of phosphofruttokinase-1 (PFK1; panel b), glyceraldehyde 3-phosphate dehydrogenase (GAPDH; panel c), enolase (panel d), pyruvate kinase (PK; panel e), lactate dehydrogenase (LDH; panel **f.**) was measured in cell lysates. Data are presented as means ± SD (*n* = 3). For all panels, versus untreated A549 cells: *p < 0.05; versus untreated A549/MDR cells: °*p* < 0.002. **g.** The glucose flux through glycolysis and TCA cycle was measured in the whole cell radiolabelled with [6-^14^C]-glucose. Data are presented as means ± SD (*n* = 3). Versus untreated A549 cells: *p < 0.01; versus untreated A549/MDR cells: °*p* < 0.002. **h.** ATP levels were measured in living cells by a chemiluminescence-based assay. Data are presented as means ± SD (*n* = 3). Versus untreated A549 cells: *p < 0.001; versus untreated A549/MDR cells: °*p* < 0.002.

In keeping with the higher expression of the glycolytic genes, A549/MDR cells showed higher uptake of glucose (Figure 3a), higher activity of PFK-1 (Figure 3b), GAPDH (Figure 3c), enolase (Figure 3d), PK (Figure 3e) and LDH (Figure 3f), higher flux of glucose into the tricarboxylic acid (TCA) cycle (Figure 3g), higher levels of ATP (Figure 3h). NZ significantly reduced all these parameters more efficiently than ZA. Again NZ was more effective in A549/MDR cells than in A549 cells (Figure 3a–3h).

### NZ inhibits the mitochondrial metabolism and increases the reactive oxygen species production in MDR cells

**Figure 4:**
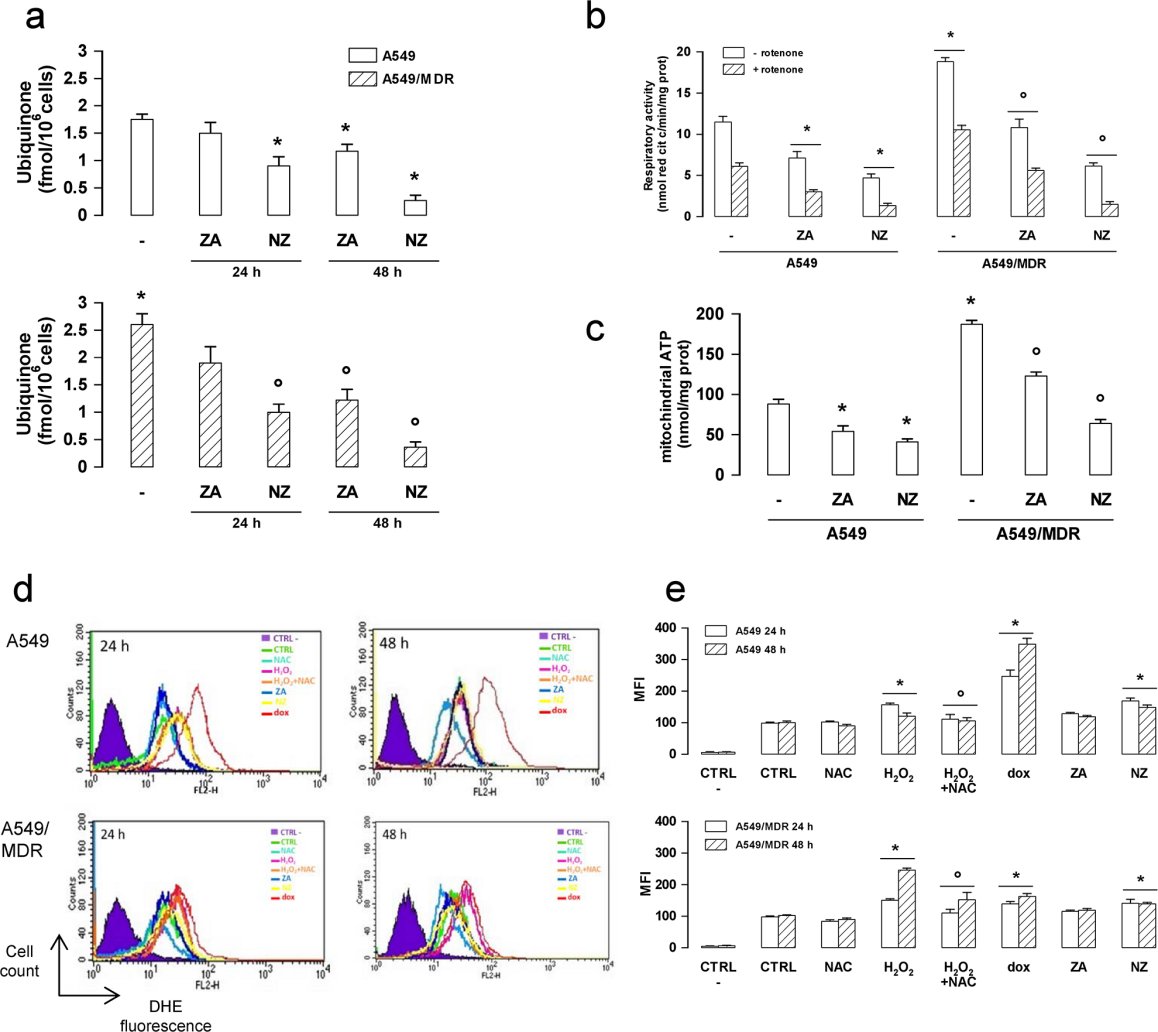
NZ reduces the mitochondrial respiratory chain activity and increases ROS in MDR cancer cells Chemosensitive human lung cancer A549 cells and their resistant counterpart A549/MDR cells were grown for 24 (panel a, d, e) or 48 h (panel a–e) in fresh medium (−), in medium containing 1 μM zoledronic acid (ZA) or 1 μM self-assembling ZA formulation (NZ). **a.** Cells were radiolabelled during the last 24 h with [^3^H]-acetate, then the *de novo* synthesis of ubiquinone was measured. Data are presented as means ± SD (*n* = 3). Versus untreated A549 cells: *p < 0.05; versus untreated A549/MDR cells: °*p* < 0.005. **b.** The ubiquinone-independent (i.e. without Complex I inhibitor rotenone) and ubiquinone-dependent (i.e. with 50 μM rotenone) electron flux between Complex I and III was measured spectrophotometrically in isolated mitochondria. Data are presented as means ± SD (*n* = 3). Versus untreated A549 cells: *p < 0.01; versus untreated A549/MDR cells: °*p* < 0.001. **c.** ATP levels were measured in mitochondrial extracts by a chemiluminescence-based assay. Data are presented as means ± SD (*n* = 3). Versus untreated A549 cells: *p < 0.05; versus untreated A549/MDR cells: °*p* < 0.001. **d.** ROS levels were measured by flow cytometry with the DHE method. H_2_O_2_ (500 μM), a ROS inducer in both chemosensitive and MDR cells, doxorubicin (1 μM, dox), a ROS inducer in chemosensitive but not in MDR cells, N-acetylcysteine (2 mM, NAC), an antioxidant agent, were used as internal controls. CTRL -: cells not labelled with DHE, used as negative control. CTRL: cells labelled with DHE, used as control of the cell autofluorescence. The figure is representative of 3 experiments. **e.** Mean fluorescence intensity (MFI) obtained in the flow cytometry experiments of panel d. Data were calculated with the Cell Quest software and are presented as means ± SD (*n* = 3). Versus CTRL: *p < 0.05; H_2_O_2_ + NAC versus H_2_O_2_ alone: °*p* < 0.05.

The synthesis of ubiquinone, whose isoprenoid tail is a side product of the mevalonate pathway, was higher in A549/MDR cells than in A549 cells (Figure 4a). The higher amount of ubiquinone was paralleled by the higher activity of the respiratory chain (Figure 4b) and by the higher level of mitochondrial ATP (Figure 4c). NZ and ZA reduced the electron flux and the ATP levels proportionally to their ability to decrease ubiquinone (Figure 4a–4c).

NZ also increased the reactive oxygen species (ROS) in A549 and A549/MDR cells (Figure 4d–4e).

### NZ reduces the efflux activity of ABC transporters and increases the intracellular drug retention in MDR cells

**Figure 5:**
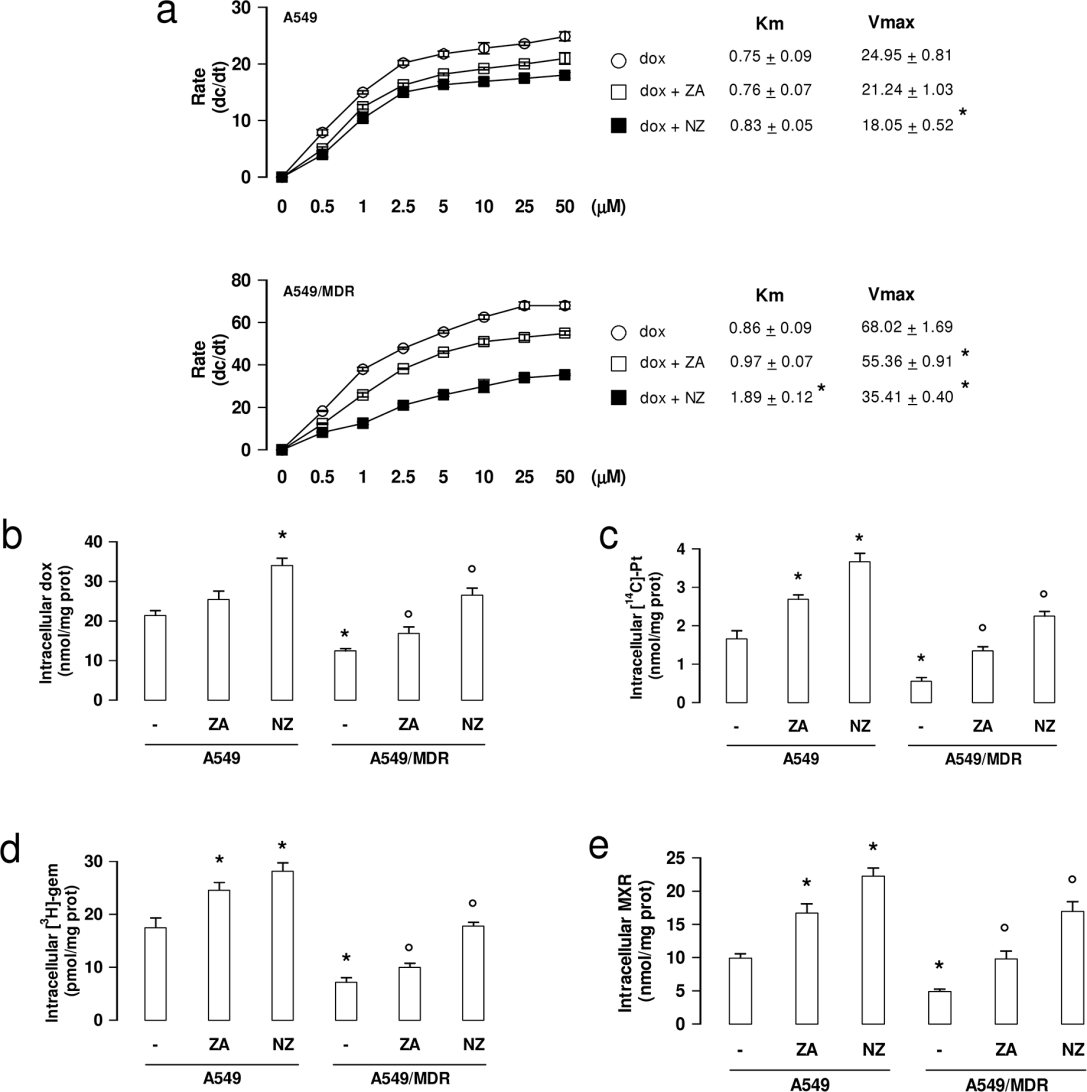
NZ reduces the efflux and increases the intracellular retention of chemotherapeutic drugs **a.** Chemosensitive human lung cancer A549 cells and their resistant counterpart A549/MDR cells were grown for 48 h in fresh medium, then incubated for 20 min with increasing concentrations (0–50 μmol/l) of doxorubicin (dox). When indicated, cells were pre-treated for 48 h with 1 μM zoledronic acid (ZA) or 1 μM self-assembling ZA formulation (NZ), then incubated with doxorubicin (dox + ZA or dox + NZ, respectively). Cells were washed and tested for the intracellular drug content. The procedure was repeated on a second series of dishes, incubated in the same experimental conditions and analyzed after 10 min. Data are presented as means ± SD (*n* = 3). The rate of doxorubicin efflux (dc/dt) was plotted versus the initial concentration of the drug. Vmax (nmol/min/mg proteins) and Km (nmol/mg proteins) were calculated with the Enzfitter software. Versus dox: *p < 0.05. **b–e.** Cells were cultured for 48 h in fresh medium (−), in medium containing 1 μM zoledronic acid (ZA) or 1 μM self-assembling ZA formulation (NZ), then incubated for 3 h with 5 μM doxorubicin (dox; panel b), 1 μCi [^14^C]-carboplatin (Pt; panel c), 0.5 μCi [^3^H]-gemcitabine (gem; panel d), 10 μM mitoxantrone (MXR; panel e). Cells were lysed and the amount of each drug was measured. Data are presented as means ± SD (*n* = 4). For all panels, versus untreated A549 cells: *p < 0.05; versus untreated A549/MDR cells: °*p* < 0.05.

Since doxorubicin is a substrate of Pgp, MRP1, MRP2, MRP3 and BCRP [6], we measured its efflux kinetics as a sensitive index of the activity of these transporters. As expected, A549/MDR cells had a higher Vmax of doxorubicin efflux than A549 cells (Figure 5a). Neither ZA nor NZ changed the Vmax in A549 cells (Figure 5a). By contrast, NZ and – at lesser extent – ZA decreased the Vmax in A549/MDR cells (Figure 5a). Moreover NZ increased the doxorubicin Km, suggesting that it reduced the affinity of the drug for the transporters. The intracellular accumulation of doxorubicin, carboplatin (a substrate of MRP1, MRP2, MRP4), gemcitabine (a substrate of MRP5) and mitoxantrone (a substrate of BCRP, Pgp, MRP1) were all significantly increased by NZ in A549/MDR cells (Figure 5b–5e).

### The chemosensitizing effect of NZ is due to the inhibition of FPPS

The chemosensitizing properties of NZ were not due to the use of the NPs scaffold: indeed blank NPs did not reduce the activity of mevalonate pathway, the activation of HIF-1α, the levels of ATP, the transcription of *Pgp* and the efflux of doxorubicin in A549/MDR cells (Supplementary Figure 4a–4e).

**Figure 6:**
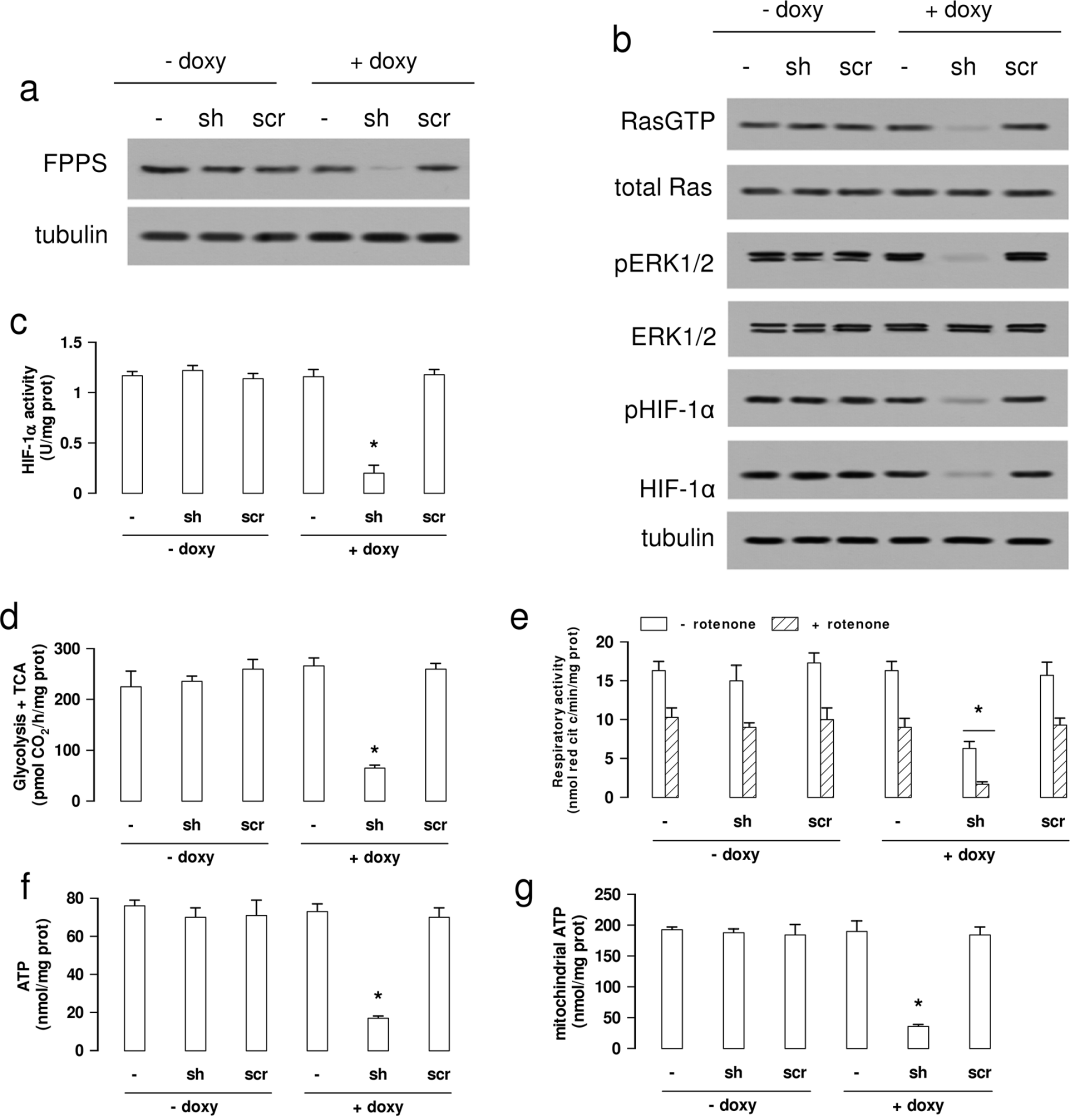
FPPS silencing produces the same metabolic signature of NZ in MDR cells Wild-type (−) A549/MDR cells, cells treated with a TetON vector containing a shRNA targeting *FPPS* (sh) or a non targeting scrambled shRNA (scr), were cultured 48 h in medium without (−) or with (+) 1 μg/ml doxycycline (doxy). **a.** Western blot analysis of FPPS. The β-tubulin expression was used as control of equal protein loading. The figure is representative of 3 experiments. **b.** Western blot analysis for Ras-GTP, Ras, phospho(Thr202/Tyr204, Thr185/Tyr187)-ERK1/2, ERK1/2, phospho(Ser)-HIF-1α, HIF-1α. The β-tubulin expression was used as control of equal protein loading. The figure is representative of 3 experiments. **c.** HIF-1α activity was measured in nuclear extracts by ELISA. Data are presented as means ± SD (*n* = 4). Versus untreated (− doxy) cells: *p < 0.001. **d.** The glucose flux through glycolysis and TCA cycle was measured by metabolic radiolabelling. Data are presented as means ± SD (*n* = 3). Versus untreated (− doxy) cells: *p < 0.001. **e.** The ubiquinone-independent (i.e. without Complex I inhibitor rotenone) and ubiquinone-dependent (i.e. with 50 μM rotenone) electron flux between Complex I and III was measured spectrophotometrically in isolated mitochondria. Data are presented as means ± SD (*n* = 3). Versus untreated (− doxy) cells: *p < 0.005. **f–g.** Whole cell (panel f) and mitochondrial (panel g) ATP was measured by a chemiluminescence-based assay. Data are presented as means ± SD (*n* = 3). For both panels, versus untreated (− doxy) cells: *p < 0.001.

To investigate whether the properties of NZ were due to the inhibition of the ZA-target enzyme FPPS, we produced a A549/MDR subclone inducibly knocked-down for *FPPS* (Figure 6a). As expected, *FPPS*-silenced A549/MDR cells had extremely low levels of cholesterol, FPP and ubiquinone (Supplementary Figure 5a–5c). Moreover, they had lower activity of Ras and ERK1/2 (Figure 6b), lower phosphorylation (Figure 6b) and DNA binding of HIF-1α (Figure 6c), lower glucose flux into glycolysis and TCA cycle (Figure 6d), lower mitochondrial respiratory activity (Figure 6e), lower levels of total (Figure 6f) and mitochondrial (Figure 6g) ATP, reproducing the same metabolic effects of NZ.

*FPPS*-silenced A549/MDR cells were also more sensitive to several cytotoxic drugs than parental A549/MDR cells (Table 3), as it occurred for A549/MDR cells treated with NZ.

**Table 3:** IC_50_ (μM) of different chemotherapeutic drugs in A549/MDR cells inducibly silenced for *FPPS*

		− doxy	− doxy	− doxy	+ doxy	+ doxy	+ doxy
Drug	Transporter	CTRL	shRNA FPPS	scrambled	CTRL	shRNA FPPS	scrambled
**doxorubicin**	Pgp, MRP1, MRP2, MRP3, BCRP	1.91 ± 0.15	1.80 ± 0.08	1.74 ± 0.17	1.81 ± 0.13	0.50 ± 0.15*	1.87 ± 0.21
**vinblastine**	Pgp, MRP1, MRP2	10.71 ± 1.01	10.22 ± 0.98	12.09 ± 1.09	11.88 ± 0.41	0.78 ± 0.18*	10.72 ± 0.35
**etoposide**	Pgp, MRP1, MRP2, MRP3	9.12 ± 0.56	8.81 ± 0.27	9.01 ± 0.71	9.01 ± 0.59	0.31 ± 0.04*	8.36 ± 0.61
**irinotecan**	Pgp, MRP1, MRP2	8.92 ± 0.62	9.07 ± 0.45	7.99 ± 0.56	8.76 ± 0.51	1.01 ± 0.21*	9.02 ± 0.49
**cisplatin**	MRP1, MRP2, MRP4	11.67 ± 0.61	10.98 ± 0.41	11.78 ± 0.91	11.23 ± 0.81	0.22 ± 0.0*	12.12 ± 0.67
**oxaliplatin**	MRP1, MRP4	4.92 ± 0.34	4.31 ± 0.27	5.11 ± 0.22	5.07 ± 0.27	0.57 ± 0.25*	5.11 ± 0.41
**5-fluorouracile**	MRP1, MRP3, MRP4, MRP5	7.22 ± 0.09	7.31 ± 0.21	8.00 ± 0.39	6.88 ± 0.26	1.39 ± 0.21*	7.01 ± 0.62
**methotrexate**	MRP4, Pgp, MRP1, MRP2, MRP3, BCRP	9.61 ± 0.71	8.99 ± 0.41	9.16 ± 0.17	8.81 ± 0.24	0.71 ± 0.13*	9.31 ± 0.38
**pemetrexed**	MRP5	10.93 ± 0.62	10.23 ± 0.81	10.91 ± 0.42	11.08 ± 0.54	0.13 ± 0.04*	11.25 ± 0.54
**gemcitabine**	MRP5	0.77 ± 0.11	0.84 ± 0.09	0.81 ± 0.07	0.91 ± 0.04	0.05 ± 0.01*	0.82 ± 0.12
**mitoxantrone**	BCRP, Pgp, MRP1	19.02 ± 0.71	18.11± 0.91	17.99 ± 0.42	18.28 ± 0.72	0.27 ± 0.03*	18.56 ± 0.81

Untreated (CTRL) A549/MDR cells, cells treated with the shRNA targeting *FPPS* (shRNA FPPS) or with a non targeting scrambled shRNA (scrambled), in the absence (−) or presence (+) of 1 μg/ml doxycycline (doxy), were incubated for 72 h with increasing concentrations of chemotherapeutic drugs, then stained in quadruplicate with neutral red (*n* = 3). Versus - doxy: *p < 0.001.

### NZ reverses drug resistance in human lung cancer xenografts

**Figure 7:**
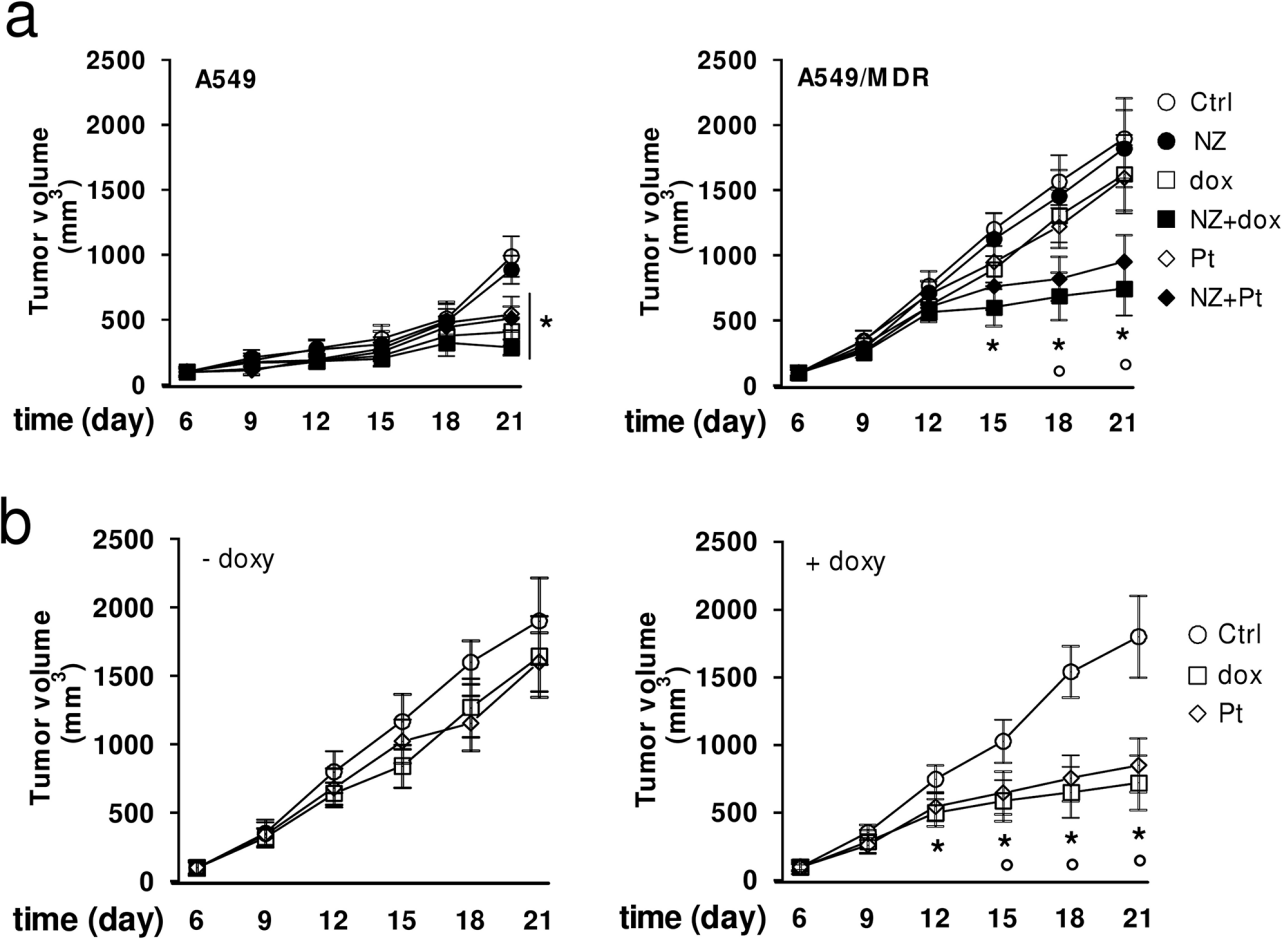
NZ reverts chemoresistance in lung xenografts **a.** Six weeks-old female BALB/c mice bearing a 100 mm^3^ A549 or A549/MDR tumor were randomly divided into 6 groups (5 mice/group) and treated with saline solution (Ctrl), NZ, doxorubicin (dox), NZ + doxorubicin (NZ+dox), carboplatin (Pt), NZ + carboplatin (NZ + Pt), as detailed under Materials and Methods. The experiment was repeated 2 times. For A549 tumors, all treatment groups versus Ctrl group (day 21): *p < 0.01. For A549/MDR tumors, NZ+dox/NZ+Pt versus Ctrl group: *p < 0.01; NZ+dox/NZ+Pt versus dox or Pt group: °*p* < 0.005. **b.** Six weeks-old female BALB/c mice bearing a 100 mm^3^ A549/MDR tumor inducibly silenced for *FPPS* were randomly divided into 3 groups (5 mice/group): Ctrl group; doxorubicin group (dox); carboplatin group (Pt), treated as detailed under Materials and Methods, without (−) or with (+) 1 mg/ml doxycycline (doxy) in the drinking water. The experiment was repeated 2 times. Dox/Pt group versus Ctrl -doxy group: *p < 0.005; dox/Pt group versus Ctrl +doxy group: °*p* < 0.01.

In keeping with the *in vitro* results, doxorubicin and carboplatin reduced the growth of A549 xenografts, but not of A549/MDR ones. NZ rescued the antitumor efficacy of these chemotherapeutic drugs in MDR tumors (Figure 7a). Of note, NZ did not increase liver, heart and kidney toxicity, as suggested by the hematochemical parameters of the animals (Supplementary Table 2). Similarly to what observed in NZ-treated mice, the *FPPS*-silencing rescued the efficacy of doxorubicin and carboplatin in A549/MDR xenografts (Figure 7b).

## DISCUSSION

In this work we investigated the potential use of self-assembling NPs encapsulating ZA, here named NZ, as not toxic metabolic modifiers and inducers of collateral sensitivity against human MDR cells.

NZ decreased the expression of Pgp, without changing the expression of other ABC transporters, but it chemosensitized MDR cells also to cytotoxic agents that are not Pgp substrates. Such mechanism is extremely surprising in the field of chemosensitizing agents, because up-to-day most chemosensitizer compounds inhibit one or few specific ABC transporters [16, 26].

By decreasing the synthesis of cholesterol, which is critical for the activity of Pgp [7, 8], and the activity of Ras/ERK1/2/HIF-1α-axis, which mediates the transcription of *Pgp* [8], NZ reversed the resistance towards Pgp substrates.

The chemosensitization towards cytotoxic agents that are not substrates of Pgp was due to the effects on the energy metabolism of MDR cells.

Many HIF-1α-target genes involved in the glycolytic flux were up-regulated in MDR cells if compared with chemosensitive ones. This condition, which is compatible with the Warburg effect observed in many solid tumors, increased the glucose flux through the glycolysis and TCA cycle, and the intracellular levels of ATP. Also the electron flux through the mitochondrial respiratory chain and the mitochondrial synthesis of ATP were increased in MDR cells. Such increase can be explained by the higher supply of reducing equivalents through the accelerated TCA cycle and/or by the higher levels of the electron shuttle ubiquinone, which is a side product of the mevalonate pathway. Chemoresistant cells often activate both glycolysis and oxidative phosphorylation to ensure an adequate supply of ATP [27], which is constantly hydrolyzed by ABC transporters. This observation is in keeping with the metabolic profile of our MDR cells, which had higher activity of both anaerobic and aerobic energy pathways and higher expression of ABC transporters than chemosensitive cells.

On the other hand, MDR cells show a paradoxical hypersensitivity - the so called “collateral sensitivity” - to agents lowering ATP or inducing oxidative stress [14, 15].

Our work suggests that NZ is a strong inducer of collateral sensitivity: it reduced glucose anaerobic and aerobic metabolism, increased ROS production and lowered intracellular ATP, by decreasing the mevalonate pathway/Ras/ERKs/HIF-1α axis and the supply of ubiquinone to the mitochondrial respiratory chain. As a consequence, NZ reduced the ATP-dependent activity of ABC transporters in MDR cells, increased the intracellular retention and cytotoxicity of multiple chemotherapeutic agents *in vitro* and *in vivo*.

These results are in line with previous data showing that agents depleting cellular ATP [28] or lowering glucose uptake and oxidative phosphorylation [29] overcome chemoresistance. Differently from other ATP depleting agents, which are highly toxic [16], NZ chemosensitized MDR cells at a concentration (1 μM) not toxic in our animal models and compatible with the concentration of ZA found in patients [30, 31].

Interestingly, NZ was significantly more effective in MDR cells than in chemosensitive ones. By targeting the mevalonate pathway and the activity of HIF-1α, which are basally more active in chemoresistant cells, NZ exploited two metabolic features that are crucial to maintain the MDR phenotype. The linkage between the inhibition of the mevalonate pathway and the resulting chemosensitizing effects was demonstrated by *FPPS*-silenced cells, which reproduced the same phenotype of NZ-treated MDR cells.

Chemoresistant cells often activate multiple survival pathways in response to stress conditions, such as JAK/STAT3 axis, Akt/mTOR axis, peroxisome proliferator activated receptor gamma-dependent pathways and cyclooxygenase 2-dependent pathways: these redundant pro-survival pathways promote cell proliferation and inhibit apoptosis, contributing to drug resistance [32–34]. We recently observed that JAK/STAT3 axis is constitutively activated in MDR cells [35] and that ZA inhibits it by reducing the Ras/ERK1/2 activity [13]. Moreover, it has been reported that ZA effects can be mediated by peroxisome proliferator activated receptor gamma [36] and cyclooxygenase 2 [37] activity. We cannot exclude that ZA and NZ chemosensitized A549/MDR cells also by targeting some of these pathways.

The use of drugs encapsulated within liposomes has been proposed as an effective strategy to overcome the drug resistance mediated by ABC transporters [38], for the different kinetics of drug release [39, 40], the different drug intracellular distribution [40, 41], the reduction of Pgp expression and activity [41–43], the changes in the lipid environment where Pgp works [44, 45].

We excluded that the chemosensitizing effect of NZ was due to the liposomal envelope, because self-assembling NPs without ZA did not reverse the MDR phenotype. On the other hand, it is known that NZ induces greater anti-proliferative effects than free ZA on tumor cells, because the use of NPs produced a higher intratumor uptake of the aminobisphosphonate [19–24]. Also in this study, the greater efficacy of NZ over free ZA can be explained by the higher uptake of ZA when administered as NZ.

**Figure 8:**
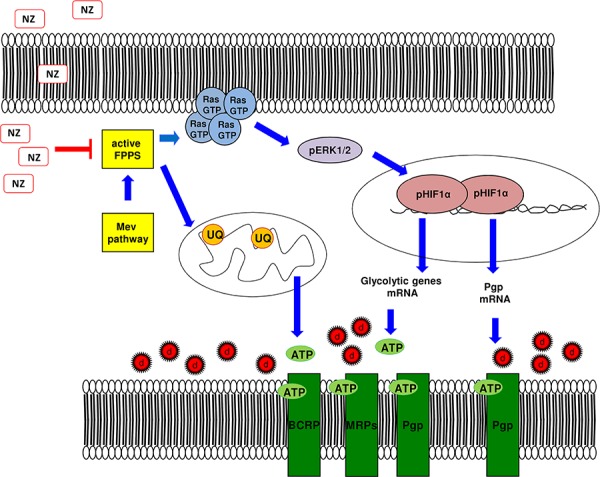
Metabolic basis of the chemosensitizing effects of NZ in MDR cells Self-assembling nanoparticles encapsulating zoledronic acid (NZ) inhibit the farnesyl pyrophosphate synthase (FPPS) step in the mevalonate (Mev) pathway. So doing, NZ reduces the Ras/ERK1/2/HIF-1α-mediated transcription of P-glycoprotein (Pgp), the transcription of glycolytic genes, the synthesis of ubiquinone (UQ) and the mitochondrial electron flux, thus limiting the supply of ATP for Pgp, multidrug resistance related proteins (MRPs) and breast cancer resistance protein (BCRP). Overall these events increase the intracellular retention and toxicity of chemotherapeutic drugs (d).

Our work unveils that NPs encapsulating ZA reverse the MDR phenotype by inhibiting the mevalonate pathway and the HIF-1α-dependent signaling, two events that impair the energy metabolism and the activity of ABC transporters (Figure 8). These observations may pave the way to the pre-clinical use of NZ, in combination with other cytotoxic drugs, as the first not toxic metabolic modifier, effective against MDR tumors.

## MATERIALS AND METHODS

### Ethics statement

Investigation has been conducted in accordance with the ethical standards, according to the Declaration of Helsinki, national and international guidelines, and has been approved by the authors' institutional review board.

### Chemicals

Fetal bovine serum and culture medium were from Invitrogen Life Technologies (Carlsbad, CA). Plasticware for cell cultures was from Falcon (Becton Dickinson, Franklin Lakes, NJ). Dihydroethidium (DHE), N-acetylcysteine (NAC) and H_2_O_2_ were purchased from Sigma-Aldrich (Milan, Italy). ZA was a gift from Novartis (Basel, Switzerland). Electrophoresis reagents were obtained from Bio-Rad Laboratories (Hercules, CA). The protein content of cell monolayers and lysates was assessed with the BCA kit from Sigma-Aldrich. Unless otherwise specified, all the other reagents were purchased from Sigma-Aldrich.

### Preparation and characterization of NZ

Self-assembling NPs encapsulating ZA were prepared as previously reported [20]. Briefly, an aqueous solution of 18 mM CaCl_2_ was added, dropwise and under magnetic stirring, to an aqueous solution of 10.8 mM Na_2_HPO_4_. The resulting suspension (termed CaPNPs) was filtered through a 0.22 μm polycarbonate filter (MF-Millipore, Microglass Heim, Italy) and stored at 4°C before use. ZA was then complexed with CaPNPs (to obtain CaPZNPs), at a volume ratio of 50:1, with a final ZA concentration of 50 mg/ml. Cationic liposomes (N-[1-(2,3-dioleoyloxy)propyl]-N,N,N-trimethylammonium chloride/cholesterol/1,2-distearoyl-*sn*-glycero-3-phosphoethanolamine-N-[amino(polyethylene glycol)-2000] at a ratio of 1:1:0.5) were prepared by hydration of a thin lipid film followed by extrusion. The lipid mixture dissolved in chloroform/methanol (2:1 v/v) was added to a 50 ml round-bottom flask and the solvent was removed under reduced pressure by a rotary evaporator (Laborota 4010 digital, Heidolph, Schwabach, Germany) in nitrogen atmosphere. The resulting lipid film was hydrated with 1 ml of 0.22 μm-filtered distilled water and the resulting suspension was gently mixed in the presence of glass beads followed by incubation at room temperature for 2 h. The liposome suspension was then extruded using a thermobarrel extruder system (Northern Lipids Inc., Vancouver, BC, Canada) passing repeatedly the suspension under nitrogen atmosphere through polycarbonate membranes with decreasing pore sizes from 400 to 100 nm (Nucleopore Track Membrane 25 mm, Whatman, Brentford, UK). The liposomes were stored at 4°C. Each formulation was prepared in triplicate. Finally, equal volumes of suspensions of the liposomes and CaPZNPs, respectively, were mixed in a glass tube and the resulting dispersion was maintained at room temperature for 10 min. NPs without ZA (blank NPs) were also prepared similarly, starting from CaPNPs and cationic liposomes. Each formulation was prepared in triplicate.

The mean diameter of stealth liposomes and CaPZNPs were determined at 20°C by photon correlation spectroscopy (N5, Beckman Coulter, Miami, FL). Each sample was diluted in deionizer/filtered water and analyzed with detector at 90° angle. P.I. was used as measure of the particle size distribution. For each batch, the mean diameter and size distribution were the mean of three measures. For each formulation, the mean diameter and P.I. were calculated as the mean of three different batches. The zeta potential (ζ) of the NPs surface was measured in water by means of a Zetasizer Nano Z (Malvern, UK). Data of ζ were collected as the average of 20 measurements.

### Encapsulation efficiency of ZA

ZA dosage was carried out as previously reported [20]. 1 ml of NPs dispersion was ultra-centrifuged (Optima Max E, Beckman Coulter) at 80,000 x g at 4°C for 40 min. Supernatants were carefully removed and ZA concentration was determined by high pressure liquid chromatography. The ZA encapsulation efficiency into CaPZNPs was calculated as [(TS_ZA_ – AS_ZA_)/TS_ZA_] × 100, where TS_ZA_ is the theoretical ZA in the supernatant and AS_ZA_ is the actual ZA concentration in the supernatant. For each formulation, the results are the mean of measures on three different batches.

### Cells

Human chemosensitive non-small cell lung cancer A549 cells were cultured in HAM's F12 medium supplemented with 10% fetal bovine serum, 1% penicillin-streptomycin and 1% L-glutamine, and were maintained in a humidified atmosphere at 37°C and 5% CO_2_. A549/MDR cells were generated by culturing parental A549 cells in medium containing increasing concentrations of doxorubicin for 30 passages and then maintaining cells at a final concentration of 100 nM doxorubicin. HT29 and HT29/MDR cells have been already described [8, 46].

### Cytotoxicity assay

Cell viability was measured by the neutral red staining method as previously reported [46]. The absorbance of untreated cells was considered as 100% viability; the results were expressed as percentage of viable cells versus untreated cells. IC_50_ was considered the concentration of each drug that reduces to 50% the cell viability versus untreated cells.

### Mevalonate pathway activity

Cells were labelled with 1 μCi/ml [^3^H]-acetate (3600 mCi/mmol; Amersham Bioscience, Piscataway, NJ). The synthesis of radiolabelled cholesterol, FPP and ubiquinone was measured as described in [8, 47]. Results were expressed as fmol [^3^H]cholesterol or [^3^H]FPP or [^3^H]ubiquinone/10 ^6^ cells, according to the relative calibration curve.

### Ras activation assay

The Ras GTP-bound fraction, taken as an index of active Ras, was measured using a pull-down assay with the Raf-1-GST fusion protein, agarose beads-conjugates (Millipore, Billerica, MA). The immunoprecipitated samples were probed with an anti-Ras (Millipore) antibody.

### Western blot

Cells were rinsed in lysis buffer (125 mM Tris-HCl, 750 mM NaCl, 1% v/v NP40, 10% v/v glycerol, 50 mM MgCl_2_, 5 mM EDTA, 25 mM NaF, 1 mM NaVO_4_, 10 μg/ml leupeptin, 10 μg/ml pepstatin, 10 μg/ml aprotinin, 1 mM phenylmethylsulfonyl fluoride, pH 7.5), sonicated and centrifuged at 13,000 x g for 10 min at 4°C. 20 μg cell lysates were subjected to Western blotting and probed with the following antibodies: phospho-(Thr202/Tyr204, Thr185/Tyr187)-ERK1/2 (Millipore); ERK1/2 (Millipore); HIF-1α (BD Bioscience, San Jose, CA); FPPS (Abcam, Cambridge, UK); Pgp/ABCB1 (Santa Cruz Biotechnology Inc., Santa Cruz, CA); MRP1/ABCC1 (Abcam); MRP2/ABCC2 (Abcam); MRP3/ABCC3 (Santa Cruz Biotechnology Inc.); MRP4/ABCC4 (Abcam); MRP5/ABCC5 (Santa Cruz Biotechnology Inc.); BCRP/ABCG2 (Santa Cruz Biotechnology Inc.); β-tubulin (Santa Cruz Biotechnology Inc.), followed by the secondary peroxidase-conjugated antibodies (Bio-Rad Laboratories). Proteins were detected by enhanced chemiluminescence (PerkinElmer, Waltham, MA). To assess HIF-1α phosphorylation, the whole cell lysate was immunoprecipitated with a polyclonal anti-HIF-1α antibody (Santa Cruz Biotechnology Inc.), then resolved by SDS-PAGE and probed with a biotin-conjugated anti-phosphoserine antibody (Sigma-Aldrich), followed by polymeric streptavidin-horseradish peroxidase-conjugates (Sigma-Aldrich).

### HIF-1α activity

Nuclear proteins were extracted using the Nuclear Extract Kit (Active Motif, Rixensart, Belgium). The activity of HIF-1 was assessed with the TransAM™ HIF-1 Transcription Factor Assay Kit (Active Motif) on 10 μg nuclear proteins. Data were expressed as U absorbance/mg nuclear proteins.

### Quantitative real time-PCR (qRT-PCR)

Total RNA was extracted and reverse-transcribed using the QuantiTect Reverse Transcription Kit (Qiagen, Hilden, Germany). qRT-PCR was carried out with IQ™ SYBR Green Supermix (Bio-Rad Laboratories). Primers sequence is listed in the Supplementary Table 3. The relative quantitation of each sample was performed using the Gene Expression Quantitation software (Bio-Rad Laboratories).

### Glucose uptake, enzymatic assays and TCA cycle activity

The uptake of glucose was measured as described earlier [48] and expressed as pmol 2-deoxy-D-[^3^H]-glucose/mg cell proteins. PFK1 assay was performed according to [49]. The activities of GAPDH, enolase, LDH were measured as reported in [50]. The activity of PK was detected with the Enzymatic Assay of Pyruvate Kinase kit (Sigma-Aldrich). Results were expressed as nmol NAD^+^/min/mg cell proteins (for PFK1, enolase, PK, LDH) or nmol NADH/min/mg cell proteins (for GAPDH). The glucose flux through glycolysis and TCA cycle was measured as described in [51] and expressed as pmol CO_2_/h/mg cell proteins.

### ATP measurement

The ATP level in whole cells and mitochondria extracts was measured with the ATP Bioluminescent Assay Kit (FL-AA, Sigma-Aldrich). ATP was quantified as arbitrary light units and converted into nmol ATP/mg cell or mitochondrial proteins, according to the calibration curve previously set.

### Respiratory chain activity

Mitochondria were extracted as described earlier [52]. A 50 μl aliquot was sonicated and used for the measurement of protein content or Western blotting; the remaining part was stored at −80°C until use. To confirm the presence of mitochondrial proteins in the extracts, 10 μg of each sonicated sample was subjected to SDS-PAGE and probed with an anti-porin antibody (Abcam; data not shown). The electron flux from Complex I to Complex III was measured on 10 μg of not sonicated mitochondrial extracts, as reported in [52]. Each sample was incubated in the absence or presence of the Complex I inhibitor rotenone (50 μM), to measure the ubiquinone-independent and the ubiquinone-dependent electron flux, respectively. The reaction was followed for 5 min, using a Packard EL340 microplate reader (Bio-Tek Instruments, Winooski, VT). Results were expressed as nmol reduced cytochrome c/min/mg mitochondrial proteins.

### Oxidative stress measurement

The evaluation of ROS was detected by using DHE, a specific marker for the determination of superoxide anion [53]. Once oxidized within the cell, DHE is converted into ethidium and emits at the wavelength of 605 nm. 2 × 10^5^ cells were incubated for 1 h with 20 ng/ml DHE, trypsinized, washed twice with PBS and re-suspended in 500 μl of PBS. The dye accumulation was analyzed by a BD FACSCalibur flow cytometer (Becton Dickinson), using the FL2-channel (band pass filter: 585 nm). For each sample, 2 × 10^4^ events were acquired. H_2_O_2_, chosen as a compound that increases ROS in both chemosensitive and MDR cells; doxorubicin, chosen as a compound that increases ROS in chemosensitive but not in MDR cells; NAC, chosen as an antioxidant agent, were included as internal controls in each experiment.

### Doxorubicin efflux rate

The kinetic parameters of doxorubicin efflux were calculated as previously reported [43]. Values were fitted to Michaelis-Menten equation to calculate Vmax and Km, using the Enzfitter software (Biosoft Corporation, Cambridge, United Kingdom).

### Intracellular drug content

Cells were incubated for 3 h with 5 μM doxorubicin, 1 μCi/ml [^14^C]-carboplatin (20 Ci/mmol; Amersham Bioscience, Piscataway, NJ), 0.5 μCi/ml [^3^H]-gemcitabine (10 Ci/mmol; Moravek Biochemicals and Radiochemicals, Brea, CA), 10 μM mitoxantrone. Samples were washed twice in PBS, detached with trypsin, centrifuged at 1,300 x g for 2 min and sonicated. The amount of [^14^C]-carboplatin and [^3^H]-gemcitabine was measured using a Tri-Carb Liquid Scintillation Analyzer (PerkinElmer). Radioactivity was converted in nmol/mg cell proteins for [^14^C]-carboplatin or pmol/mg cell proteins for [^3^H]-gemcitabine, using a calibration curve previously prepared. The amount of doxorubicin and mitoxantrone was measured fluorimetrically, using a LS-5 spectrofluorimeter (PerkinElmer). Excitation and emission wavelengths were: 475 nm and 553 nm (doxorubicin); 607 nm and 684 nm (mitoxantrone). Fluorescence was converted in nmol/mg cell proteins, using a calibration curve previously set.

### *FPPS* silencing

5 × 10^4^ A549/MDR cells were transduced with 2.5 μg Tet-On pTRIPZ vector containing a specific shRNA for *FPPS* or a non targeting sequence (Thermo Scientific Open Biosystems, Waltham, MA). Transduced clones were selected by culturing cells in medium containing 0.5 μg/ml puromycin. *FPPS* shRNA was induced by adding 1 μg/ml doxycycline for 48 h. The silencing efficacy was checked by Western blot analysis.

### *In Vivo* Tumor Growth

1 × 10^6^ A549 and A549/MDR cells, re-suspended in 20 μl culture medium mixed with 20 μl Cultrex BME (Trevigen, Gaithersburg, MD), were implanted subcutaneously in 6 weeks-old female nude BALB/c mice, housed under 12 h light/dark cycle, with food and drinking provided *ad libitum*. When the tumors reached the volume of 100 mm^3^, the animals were randomized into these groups: control group (treated with 20 μl saline solution i.v. on days 2, 8, 14); NZ group (treated with 20 μg/mouse NZ i.v. on days 2, 8, 14); doxorubicin group (treated with 4 mg/kg doxorubicin i.v. on days 3, 9, 15); NZ + doxorubicin group (treated with 20 μg/mouse NZ on days 2, 8, 14 followed by 4 mg/kg doxorubicin on days 3, 9, 15); carboplatin group (treated with 50 mg/kg carboplatin i.v. on days 3, 9, 15); NZ + carboplatin group (treated with 20 μg/mouse NZ on days 2, 8, 14 followed by 50 mg/kg carboplatin on days 3, 9, 15). In a second experimental set, A549/MDR cells inducibly silenced for *FPPS* were implanted and mice were treated with doxorubicin or carboplatin as reported above. *FPPS* silencing was induced by adding doxycycline (1 mg/ml) in the drinking water. Tumor growth was measured by caliper and was calculated according to the equation: (L × W^2^)/2, where L = tumor length and W = tumor width. Mice were euthanized at day 21. The hematochemical parameters LDH, aspartate aminotransferase (AST), alanine aminotransferase (ALT), alkaline phosphatase (AP), creatine phosphokinase (CPK), creatinine were measured on 0.5 ml of blood collected immediately after mice sacrifice, using commercially available kits from Beckman Coulter Inc. (Brea, CA). The experimental procedures were approved by the Bioethics Committee (“Comitato Etico di Ateneo”) of the University of Torino, Italy.

### Statistical analysis

All data in text and figures are provided as means ± SD. The results were analyzed by a one-way analysis of variance (ANOVA) and Tukey's test. *p* < 0.05 was considered significant.

## SUPPLEMENTARY FIGURES AND TABLES


