# Supplementary material for: Self-assembling nanoparticles encapsulating zoledronic acid revert multidrug resistance in cancer cells
Source: Oncotarget. 2015 Sep 9;6(31):31461–78. doi: 10.18632/oncotarget.5058 (PMC4741618; doi:10.18632/oncotarget.5058)
Supplement: Supplementary file 1 [file oncotarget-06-31461-s001.pdf]

SUPPLEMENTARY FIGURES AND TABLES

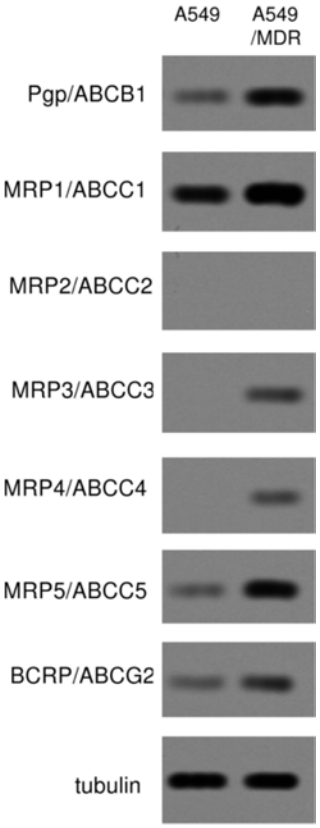

Supplementary Figure S1: Expression of ABC transporters in chemosensitive and MDR cancer cells. Chemosensitive human lung cancer A549 cells and their resistant counterpart A549/MDR cells were lysed and subjected to the Western blot analysis for Pgp/ABCB1, MRP1/ABCC1, MRP2/ABCC2, MRP3/ABCC3, MRP4/ABCC4, MRP5/ABCC5, BCRP/ABCG2. The β-tubulin expression was used as control of equal protein loading. The figure is representative of 3 experiments.

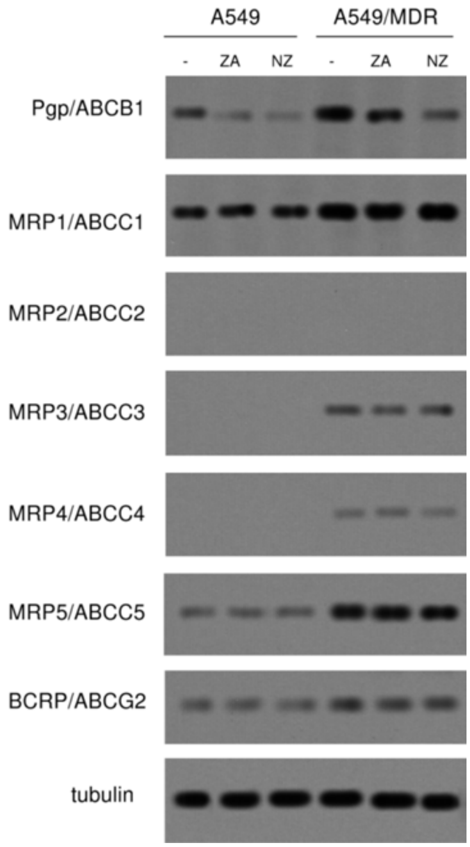

Supplementary Figure S2: Expression of ABC transporters after treatment with ZA and NZ in chemosensitive and MDR cancer cells. Chemosensitive human lung cancer A549 cells and their resistant counterpart A549/MDR cells were cultured for 48 h in fresh medium (-), in medium containing 1 µM zoledronic acid (ZA) or 1 µM self-assembling ZA formulation (NZ), then lysed and subjected to the Western blot analysis for Pgp/ABCB1, MRP1/ABCC1, MRP2/ABCC2, MRP3/ABCC3, MRP4/ABCC4, MRP5/ABCC5, BCRP/ABCG2. The β-tubulin expression was used as control of equal protein loading. The figure is representative of 3 experiments.

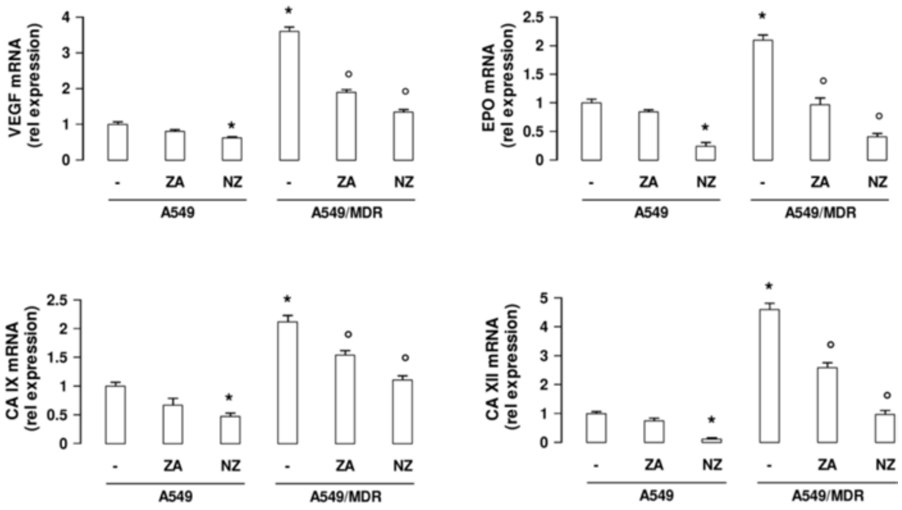

Supplementary Figure S3: Expression of HIF-1α-target genes in chemosensitive and MDR cancer cells treated with ZA and NZ. Chemosensitive human lung cancer A549 cells and their resistant counterpart A549/MDR cells were cultured for 48 h in fresh medium (-), in medium containing 1 µM zoledronic acid (ZA) or 1 µM self-assembling ZA formulation (NZ). *VEGF, EPO, CA IX, CA XII* mRNA levels were detected in triplicate by qRT-PCR. Data are presented as means ± SD ($n = 4$). For all panels, versus untreated A549 cells: *$p < 0.05$; versus untreated A549/MDR cells: °$p < 0.02$.

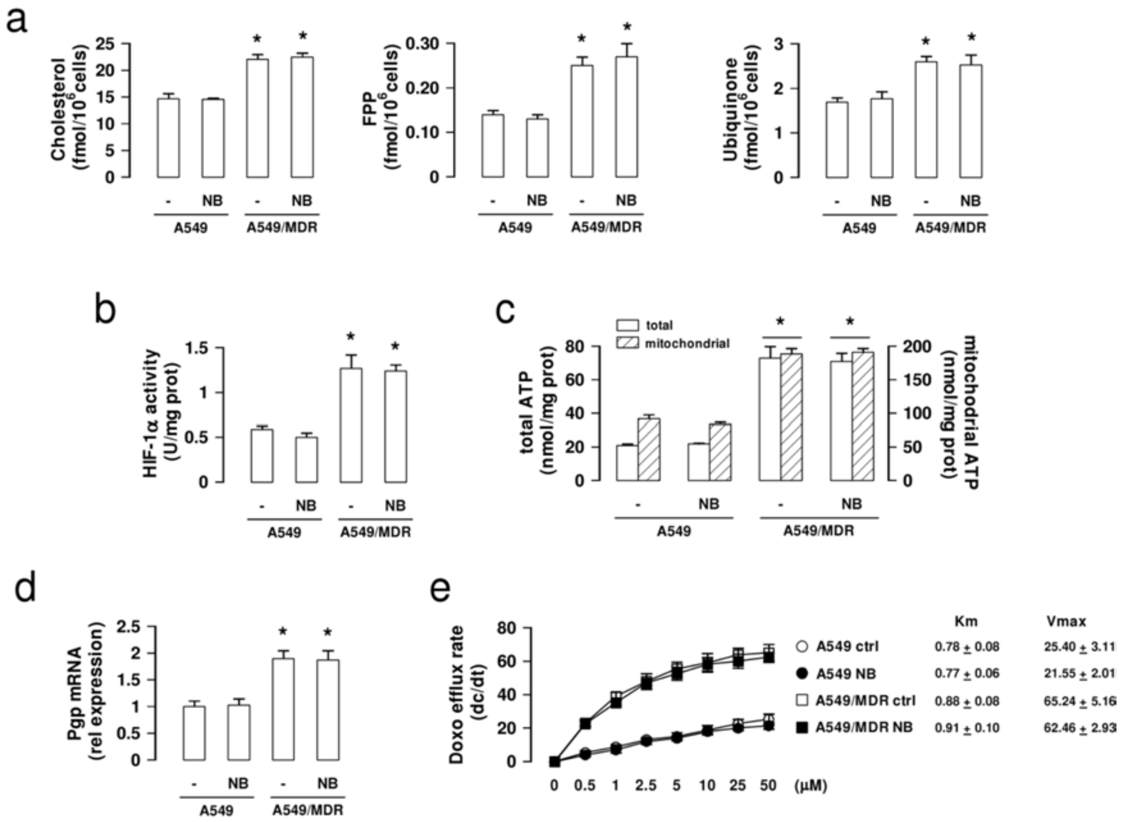

Supplementary Figure S4: Effects of empty self assembling liposomes on chemosensitive and MDR cancer cells. Chemosensitive human lung cancer A549 cells and their resistant counterpart A549/MDR cells were cultured for 48 h in fresh medium (-) or in medium containing self-assembling formulation without zoledronic acid (blank NPs, NB) at the same final concentration used in the all the other experimental assays. **a.** Cells were radiolabelled during the last 24 h with [^{3}H]-acetate, then the *de novo* synthesis of cholesterol, FPP and ubiquinone was measured. Data are presented as means ± SD ($n = 3$). For all panels, A549/MDR cells versus A549 cells: *$p < 0.02$. **b.** HIF-1α activity was measured in nuclear extracts by ELISA. Data are presented as means ± SD ($n = 3$). A549/MDR cells versus A549 cells: *$p < 0.005$. **c.** ATP levels in whole cell (total) and isolated mitochondria (mitochondrial) were measured by a chemiluminescence-based assay. Data are presented as means ± SD ($n = 3$). A549/MDR cells versus A549 cells: *$p < 0.001$. **d.** *Pgp* mRNA levels were detected in triplicate by qRT-PCR. Data are presented as means ± SD ($n = 3$). A549/MDR cells versus A549 cells: *$p < 0.001$. **e.** Cells were grown for 48 h in fresh medium or in medium containing blank NPs (NB), then incubated for 20 min with increasing concentrations (0–50 µmol/L) of doxorubicin (dox). Cells were washed and tested for the intracellular drugs content. The procedure was repeated on a second series of dishes, incubated in the same experimental conditions and analyzed after 10 min. Data are presented as means ± SD ($n = 3$). The rate of doxorubicin efflux (dc/dt) was plotted versus the initial concentration of the drug.Vmax (nmol/min/mg proteins) and Km (nmol/mg proteins) were calculated with the Enzfitter software.

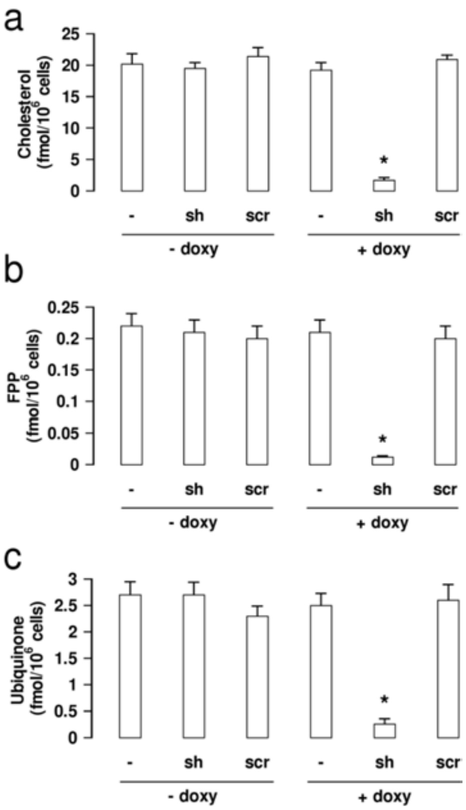

Supplementary Figure S5: Effects of FPPS silencing on the synthesis of cholesterol, FPP and ubiquinone in chemoresistant cancer cells. Wild-type A549/MDR cells, cells treated with a TetON vector containing a shRNA targeting *FPPS* (sh) or with a non targeting shRNA vector (scr), were cultured 48 h in media without (- doxy) or with (+ doxy) 1 µg/ml doxycycline. Cells were radiolabelled during the last 24 h with [^{3}H]-acetate, then the *de novo* synthesis of cholesterol (panel **a**), FPP (panel **b**) or ubiquinone (panel **c**) was measured. Data are presented as means ± SD ($n = 3$). For all panels, versus untreated (-, - doxy) cells: *$p < 0.001$.

Supplementary Table S1: IC50 (µM) of different cytotoxic drugs in HT29 and HT29/MDR cells

Drug	Transporter	HT29	HT29	HT29	HT29/MDR	HT29/MDR	HT29/MDR
		CTRL	ZA	NZ	CTRL	ZA	NZ
**doxorubicin**	Pgp, MRP1, MRP2, MRP3, BCRP	1.23 ± 0.08	0.91 ± 0.07	0.71 ± 0.1 *	2.58 ± 0.13°	1.02 ± 0.12 *	0.98 ± 0.07*
**vinblastine**	Pgp, MRP1, MRP2	3.61 ± 0.14	2.53 ± 0.17	1.88 ± 0.14 *	9.23 ± 0.41°	3.21 ± 0.16 *	0.51 ± 0.09*
**etoposide**	Pgp, MRP1, MRP2, MRP3	1.12 ± 0.15	0.67 ± 0.11 *	0.51 ± 0.06 *	11.41 ± 0.37°	2.76 ± 0.44 *	2.33 ± 0.18*
**irinotecan**	Pgp, MRP1, MRP2	6.23 ± 0.37	4.41 ± 0.31	3.71 ± 0.44 *	47.11 ± 5.14°	36.11 ± 3.71	14.23 ± 0.91*
**cisplatin**	MRP1, MRP2, MRP4	10.89 ± 1.21	7.21 ± 0.88	6.01 ± 0.13 *	52.14 ± 2.47°	5.88 ± 0.42 *	2.24 ± 0.55*
**oxaliplatin**	MRP1, MRP4	5.87 ± 0.61	2.43 ± 0.52 *	0.91 ± 0.15 *	12.21 ± 0.18°	5.21 ± 0.23 *	0.81 ± 0.16*
**5-fluorouracile**	MRP1, MRP3, MRP4, MRP5	0.83 ± 0.11	0.61 ± 0.14	0.65 ± 0.13	7.53 ± 0.71°	5.44 ± 0.23	3.12 ± 0.14*
**methotrexate**	MRP4, Pgp, MRP1, MRP2, MRP3, BCRP	2.34 ± 0.31	1.01 ± 0.18 *	0.21 ± 0.09 *	8.79 ± 0.71°	1.77 ± 0.45 *	0.46 ± 0.22*
**pemetrexed**	MRP5	0.74 ± 0.01	0.65 ± 0.17	0.12 ± 0.07 *	8.26 ± 0.57°	5.78 ± 0.56	1.33 ± 0.27*
**gemcitabine**	MRP5	0.11 ± 0.03	0.11 ± 0.09	0.06 ± 0.01 *	0.75 ± 0.08°	0.21 ± 0.09 *	0.05 ± 0.02*
**mitoxantrone**	BCRP, Pgp, MRP1	5.88 ± 0.41	2.21 ± 0.17 *	2.03 ± 0.21 *	9.67 ± 0.41°	4.53 ± 0.37 *	2.81 ± 0.41*

Untreated (CTRL) HT29 and HT29/MDR cells, cells treated with ZA or NZ (1 µM), were incubated for 72 h with increasing concentrations of cytotoxic drugs, then stained in quadruplicate with neutral red ($n = 3$). Versus respective CTRL: *$p < 0.05$; HT29/MDR versus HT29 cells: °$p < 0.001$.

Supplementary Table S2: Hematochemical parameters of animals

		LDH (U/l)	AST (U/l)	ALT (U/l)	AP (U/l)	CPK (U/l)	creatinine (mg/l)
A549	Ctrl	6342 ± 1567	267 ± 56	39 ± 12	75 ± 16	434 ± 68	0.05 ± 0.01
	NZ	6872 ± 1812	293 ± 24	46 ± 11	87 ± 24	511 ± 76	0.06 ± 0.02
	dox	7862 ± 2089	254 ± 34	41 ± 13	81 ± 23	897 ± 71*	0.05 ± 0.02
	NZ+dox	6723 ± 1629	309 ± 31	50 ± 22	73 ± 27	911 ± 56*	0.06 ± 0.03
	Pt	6341 ± 1098	311 ± 24	45 ± 17	76 ± 23	467 ± 71	0.09 ± 0.01*
	NZ+Pt	6009 ± 1987	298 ± 66	49 ± 17	75 ± 11	509 ± 87	0.10 ± 0.02*
A549/MDR	Ctrl	7098 ± 1803	288 ± 24	47± 18	78 ± 26	454 ± 91	0.04 ± 0.02
	NZ	7612 ± 2137	291 ± 34	51 ± 16	87 ± 21	459 ± 37	0.06 ± 0.02
	dox	6534 ± 1271	254 ± 71	45 ± 16	80± 14	434 ± 68	0.04 ± 0.01
	NZ+dox	6093 ± 1234	312 ± 45	55 ± 23	80 ± 20	987 ± 99 *	0.05 ± 0.04
	Pt	6092 ± 1261	324 ± 71	56 ± 25	77 ± 23	467 ± 93	0.08 ± 0.01*
	NZ+Pt	6873 ± 1093	321 ± 81	47 ± 16	71 ± 34	489 ± 88	0.09 ± 0.02*

Six weeks old female BALB/c mice bearing a 100 mm^3-tumor of A549 or A549/MDR cells were randomly divided in the following groups (5 mice/group) and treated with saline solution (Ctrl), NZ, doxorubicin (dox), NZ + doxorubicin (NZ+dox), carboplatin (Pt), NZ + carboplatin (NZ + Pt), as detailed under Materials and Methods. The experiment was repeated 2 times. The animals were sacrificed at day 21 after randomization. Blood was collected immediately after mice euthanasia. Versus Ctrl group: $*p < 0.02$.

Supplementary Table S3: Primers sequence for qRT-PCR

Gene	Forward primer	Reverse primer
GLUT1	CCTGCAGTTTGGCTACAACA	TAACGAAAAGGCCCACAGAG
HK	AGACGCACCCACAGTATTCC	CGCATCCTCTTCTTCACCTC
PFK1	GGAGCTTCGAGAACAACTGG	CTGTGTGTCCATGGGAGATG
ALDO-A	GCTATGGCCTTTTCCTTTCC	ATGCTCCCAGTGGACTCATC
GAPDH	GAAGGTGAAGGTCGGAGT	CATGGTGGAATCATATTGGAA
PGK	TCTCATGGATGAGGTGGTGA	CTTCCAGGAGCTCCAAACTG
ENO-A	GCTCCGGGACAATGATAAGA	TCCATCCATCTCGATCATCA
PK	TGCAGTGGAGCTCAGAGAGA	GCTTCCGGTGACATAATGCT
LDH	TGGGAGTTCACCCATTAAGC	AGCACTCTCAACCACCTGCT
VEGF	ATCTTCAAGCCATCCTGTGTGC	GCTCACCGCCTCGGCTTGT
EPO	CAGACTTCTACGGCCTGCTG	GCTGAACACTGCAGCTTGAA
CA IX	GTCTCGCTTGGAAGAAATCG	AGAGGGTGTGGAGCTGCTTA
CA XII	ACTGAGTCTCTGGGCATCATCC	AAAAGCCAAATGGACACCAC
Pgp	TGCTGGAGCGGTTCTACG	ATAGGCAATGTTCTCAGCAATG
S14	GGTGCAAGGAGCTGGGTAT	TCCAGGGGTCTTGGTCCTATTT
